# Cholesterol Metabolic Markers for Differential Evaluation of Patients with Hyperlipidemia and Familial Hypercholesterolemia

**DOI:** 10.1155/2022/2008556

**Published:** 2022-04-21

**Authors:** Zhi-Zhao Li, Qiong Huang, Xiao-li Yang, Jieqiong Zeng, QI-Hui Wang, Hai-Ming Tang, Zhen-qiu Yu, Yu-Qing Song, Yang Liu

**Affiliations:** ^1^Beijing DiTan Hospital, Capital Medical University, Beijing 100015, China; ^2^Beijing Anzhen Hospital, Capital Medical University, Beijing Institute of Heart Lung and Blood Vessel Diseases, Beijing 100029, China; ^3^Hunan Polytechnic of Environment and Biology, Hengyang 421005, China; ^4^Shouguang People's Hospital of Shandong Province, Shouguang 262700, China; ^5^Beijing Changping Hospital of Chinese and Western Medicine, Beijiing 102206, China; ^6^Sinochem Environment Holdings Co, Ltd, Beijing 100045, China; ^7^Beijng Academy of Science and Technology, Beijing 100089, China; ^8^NO.922 Hospital OF PLA Joint Logistics Support Force, Hengyang 421002, China

## Abstract

The cholesterol metabolism in humans can be indirectly reflected by measuring cholesterol metabolism marker levels. We aimed to investigate the association of cholesterol homeostasis markers on standard lipid profiling components in familial hypercholesteremia and hyperlipidemia patients. A total of 69 hyperlipidemia patients, 25 familial hypercholesteremia (FHC) patients, and 64 healthy controls were enrolled in this study. We performed routine testing of blood lipid water. Gas chromatography was used to determine the changes in the concentration of cholesterol synthesis (squalene, desmosterol, and lathosterol) and absorption markers (campesterol, sitosterol, and stigmasterol) in the blood. Baseline hyperlipidemia patients displayed significantly higher total cholesterol (TC), triglyceride (TG), and low-density lipoprotein cholesterol (LDL-C) levels in comparison to the control group, which was reflected in the increased levels of squalene, desmosterol, campesterol, and sitosterol observed (*P* < 0.05) in the hyperlipidemia patients. The desmosterol, lathosterol, campesterol, stigmasterol, and sitosterol were statistically different in the FHC group than the hyperlipidemic group (*P* < 0.05). The proportions of squalene/cholesterol, lathosterol/cholesterol, stigmasterol/cholesterol, and sitosterol/cholesterol in the FHC group were lower than those in the hyperlipidemic group; only desmosterol/cholesterol was higher than that in the hyperlipidemic group. Correlation studies between lipid metabolic factors showed that the proportion of moderate and strong correlations was much higher in the FHC group than in the other two groups (76.92% vs. 32.50% and 31.25%). Logistic regression analysis showed that the concentrations of glucose, LDL-C, lactosterol, and sitosterol were all independent risk factors for developing hyperlipidemia. This result was further confirmed by the ROC curve. These results indicated that the study of cholesterol synthesis and decomposition markers can serve as a reference index for related diseases caused by changes in its concentration.

## 1. Introduction

Hyperlipoidemia refers to the blood cholesterol and triglyceride concentrations exceeding the normal range, which can cause some diseases that seriously endanger human health [1, 2]. Imbalance in cholesterol metabolism is one of the characteristics of hyperlipidemia [3]. Cholesterol homeostasis is attained via its synthesis and absorption in the gastrointestinal tract [4]. Studies have shown that small, dense LDL is an independent risk factor for cardiovascular diseases [5]. However, the relative proportion of small, dense LDL was higher in high cholesterol synthesis compared to low synthesis; desmosterol to *β*-sitosterol ratio could be used as evaluating individual propensity toward dyslipidemia development and direct the future treatment [6]. At present, although there are many reports on the levels of cholesterol absorption and synthetic markers, there are very few studies reflecting hyperlipidemia and FHC patients [7, 8]. This study performed gas chromatography tests in patients with hyperlipidemia, familial hypercholesterolemia, and healthy controls to determine changes in cholesterol uptake and synthesis markers, to understand how these markers correlate with lipid indices, and to further evaluate these markers to complement the identification and treatment of patients with hyperlipidemia and FHC.

## 2. Materials and Methods

### 2.1. Study Participants

A total of 69 hyperlipidemia patients, including 30 male patients and 39 female patients, were selected from the outpatient department of Beijing Anzhen Hospital, Capital Medical University. The mean age of the hyperlipidemia patients was 50.3 ± 5.8 years. The inclusion criteria were total cholesterol (TC) > 5.7 mmol/L and/or triglyceride (TG) > 1.7 mmol/L, while the exclusion criteria were the presence of any liver, kidney, endocrine, and metabolic diseases that could affect lipid metabolism [9].

Furthermore, 25 patients with familial hypercholesteremia patients (FHC) were selected, of which 13 were males and 12 were female. The mean age among this group was 29.5 ± 23 years (median: 24 years; range 6-53 years). The healthy control group consisted of 64 healthy people, including 16 males and 48 females, whose median age was 51.0 (45-55 years), whose liver and kidney functions were deemed normal and showed absence of any diseases that could affect lipid metabolism during physical examination. The Medical Ethics Committee of Anzhen Hospital approved the study protocol and written informed consent was obtained from each patient from the hyperlipidemia, FHC, and healthy control group. Furthermore, it is to be noted that all methods were carried out following relevant guidelines and regulations.

### 2.2. Methods

All examinations were carried out in the hospital. Trained research technicians asked the study participants questions from a standard questionnaire providing information on demographic variables such as age, gender, known diagnosis of dyslipidemia, and current treatment of dyslipidemia. Hyperlipidemia was defined as any of hypercholesterolemia (total cholesterol concentration ≥ 5.72 mmol/L (220 mg/dL) or hypertriglyceridemia (triglyceride concentration ≥ 1.70 mmol/L (150 mg/dL) or lower concentration of high-density lipoprotein-cholesterol (HDL − C) ≤ 0.91 mmol/L (35 mg/dL) [10].

### 2.3. Determination of Biochemical Indexes

The subjects' non-anticoagulated, 12-hour fasting venous blood was collected. The blood of the subjects was centrifuged at 4000 rpm for 10 minutes. Blood lipids, liver function, and kidney function were measured by conventional methods. The serum concentrations of TC, TG, HDL-C, low-density lipoprotein-cholesterol (LDL-C), and fasting plasma glucose (FPG) were measured using standard enzymatic methods (Automatic biochemical analyzer, 630 Semiautomatic biochemical analyzer Crony, Roma, Italy).

### 2.4. Cholesterol Absorption and Synthetic Markers

A gas chromatography (GC) method was employed to identify cholesterol synthesis (squalene, lathosterol, and desmosterol) and absorption markers (campesterol, stigmasterol, and sitosterol). The serum was assayed by saponification with an alkaline alcohol solution, extraction with hexane, and silylation. The detection conditions included (1) an HP-5 quartz capillary column with an initial column temperature of 150°C, maintained for 3 min, and a programmed ramp rate of 30°C/min to 250°C; (2) a further ramp to 280°C at 5°C/min, maintained for 30 min; (3) a hydrogen flame ionization detector (FID) with an inlet temperature of 290°C and a pressure of 15 psi; and a nonsplit mode with a sample injection of 1 *μ*L. An Agilent 7890 gas chromatograph was used for image acquisition [6]. The markers were determined by the single internal standard curve method. As noncholesterol sterols are transported in the plasma by lipoproteins, it is common to adjust them for the total plasma cholesterol level by expressing noncholesterol sterols as a ratio to the cholesterol level (noncholesterol sterol/cholesterol).

### 2.5. Statistical Methods

All measured data were statistically analyzed using the SPSS statistical package (SPSS version 25.0, Chicago, IL, USA), with numerical data expressed as mean ± SD for the normally distributed variable. Comparison within and between FHC, hyperlipidemia, and healthy control groups was assessed using one-way variance analysis (ANOVA). The *t*-test was used for two-way comparisons of continuous data that conformed to the normal distribution; the Mann–Whitney*U* test was used when it was not normal distribution. Pearson's correlation analysis was used for the correlation analysis by the normal distribution, and Spearman's correlation analysis was used for the correlation analysis under the nonnormal distribution. It is weak correlation when Cor.<0.3; low correlation when 0.3 < Cor.<0.5; moderate correlation when 0.5 < Cor.<0.8; and high correlation when 0.8 < Cor.<1. Logistic regression analysis was used to screen the independent influence factors for lipid metabolism. *P* < 0.05 was considered statistically significant.

## 3. Results

### 3.1. Comparison of Clinical Baseline Data

In this study, there was no statistical difference for age, gender, and body mass index (BMI) among the hyperlipidemia and (all *P* value > 0.05, respectively). Although observations of any significance were not obtained in systolic blood pressure (SBP) and diastolic blood pressure (DBP) between the three groups (*P* > 0.05), the levels of LDL-C, HDL-C, TG, and TC were all significantly higher in patients with the FHC group when compared to the control and hyperlipidemia groups. Comparison between the two groups showed that all lipid indices and blood glucose levels were higher in the hyperlipidemia group than in the healthy control group (all *P* < 0.05). However, there was no statistical difference between the two groups in comparing SBP and DBP (both *P* < 0.05). More details are shown in Table 1.

### 3.2. Comparison of Cholesterol Absorption and Synthetic Markers

The levels of cholesterol metabolism markers (squalene, desmosterol, lathosterol, campesterol, stigmasterol, and sitosterol) were signed between the groups of FHC, hyperlipidemia, and control groups (all *P* < 0.05) (Table 1). The comparison between the two groups showed that the concentrations of squalene, desmosterol, and lathosterol were higher in the hyperlipidemic group than in the healthy control group (*P* < 0.05); however, the comparison of the concentrations of campesterol, stigmasterol, sitosterol between the two groups did not statistically between the hyoerlipidemic groups and the healthy control group(*P* > 0.05). In contrast with cholesterol, only lathosterol/cholesterol and stigmasterol/cholesterol were statistically different between the two groups (*P* < 0.05). The desmosterol, lathosterol, campesterol, stigmasterol, and sitosterol were statistically different in the FHC group than the hyperlipidemic group (*P* < 0.05). The proportions of squalene/cholesterol, lathosterol/cholesterol, stigmasterol/cholesterol, and sitosterol/cholesterol in the FHC group were lower than those in the hyperlipidemic group; only desmosterol/cholesterol was higher than that in the hyperlipidemic group.

### 3.3. Correlation Study of Lipid Factors and Cholesterol Metabolic Factors in Three Groups

There were 40 pairs of healthy controls with correlations between lipid and cholesterol metabolic indicators. There were 15 weak correlations, 12 low correlations, 7 moderate correlations, and 6 high correlations (Table 2). Eighty pairs of correlations were found in the hyperlipidemic group. There were 24 weak correlations, 31 low correlations, 22 moderate correlations, and 3 high correlations (Table 3). Twenty-six pairs of correlation were found in the FHC group. There were 0 of weak correlation, 6 of low correlations, 10 of moderate correlations, and 10 of high correlations (Table 4). The moderate and strong correlations were 32.50% in the healthy group, 31.25% in the hyperlipidemic group, and increased to 76.92% in the FHC group.

### 3.4. Logistic Regression Analysis of the Risk of Developing Cholesterolemia in Patients

In our etiological study of patients with hyperlipidemia using logistic regression analysis, the results showed that increased concentrations of glucose (mmol/L), LDL-C (mmol/L), lathosterol (*μ*mol/L), and sitosterol (*μ*mol/L) were all independent risk factors for the development of the disease factors (all *P* < 0.05). The OR of the four indicators were 2.213, 4.255, 8.599^∗^105, and 2492.153, respectively. More details are shown in Table 5.

### 3.5. The Diagnostic Results of the ROC Curve

**Figure 1:**
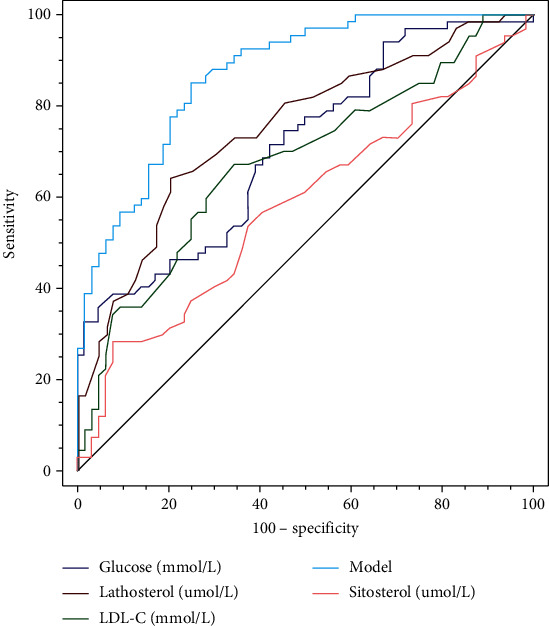
The result of the diagnostic ROC curve.

The ROC curve was used to evaluate the diagnostic efficiency of the four indicators in the logistic regression analysis. We also modelled the indicators in the logistic regression analysis. The results showed that the diagnostic efficiency of the model group was better than that of the other indicators. The AUC of the model group reached 0.871, and the Youden index was 0.601 (Figure 1). Compared to glucose (98.44) and sitosterol (92.19), which had better specificity, the model group had only moderate specificity of 75% but had better diagnostic sensitivity (85.07%). More details are shown in Table 6.

## 4. Discussion

Dyslipidemia is a risk factor for cardiovascular and cerebrovascular diseases [6]. Currently, traditional lipid indicators are used to determine cholesterol levels in clinical practice, but these indicators only reflect the final results of cholesterol metabolism. The human body's cholesterol absorption and synthesis are highly individualized, influenced by environmental and genetic factors [10–12]. As a result, the traditional lipid parameters cannot reflect the metabolic characteristics of cholesterol in the body. Cholesterol observed in the human serum is originated via either the cholesterol synthesis that occurs in the liver (endogenous cholesterol) or derived from the nourishments consumed that are absorbed in the small intestines (exogenous cholesterol), both of which contributes to cholesterol homeostasis [8, 13, 14]. Precursors of cholesterol synthesis in serum (desmosterol, lathosterol, and squalene) can be utilized as markers of cholesterol synthesis. Since the absorption rate of plant sterol (campesterol, stigmasterol, and sitosterol) is inversely proportional to cholesterol absorption, it can be indirectly used as cholesterol absorption markers [15–18]. A meta-analytic study conducted by Slibernage et al. involving 4362 subjects indicated that a marked increase in cholesterol absorption was observed in patients with cardiovascular disease [19]. The well-established Scandinavian Simvastatin Survival Study intended to analyze the impact of cholesterol-lowering treatment on the mortality and morbidity of 4444 patients with coronary heart disease (CHD). The study concluded that an increase in plasma campesterol levels as increasing the risk of CHD event reoccurrence was observed. However, these results were not reflected in the traditional lipid profiling of the patients participating in the study [20]. Residual cardiovascular disease (CVD) risk is a dilemma in clinical practice; indeed, novel lipoprotein biomarkers are suggested as possible targets for improving the outcomes of patients at higher risk for CVD [21].

At present, serum markers of cholesterol metabolism have been used in the epidemiological study of dyslipidemia metabolism; the relationship between cholesterol metabolism markers and lipid indicators is rarely reported [22]. In healthy people, cholesterol absorption and synthesis maintain a dynamic balance; when absorption increases, the synthesis of the cholesterol in the liver decreases and vice versa. These two mechanisms together maintain cholesterol homeostasis in the internal environment. This dynamic balance is disrupted when a hurdle is experienced in any two processes [23]. In this study, TC, TG, and LDL-C in hyperlipidemia patients were significantly higher than those in the healthy control group (*P* < 0.05). The concentrations of the squalene, desmosterol, and lathosterol were higher in the hyperlipidemic group than in the healthy group (*P* < 0.05); however, the concentration of the campesterol, stigmasterol, and sitosterol was not statistically different between the two groups (*P* > 0.05). The above study results showed that all three markers related to cholesterol absorption in hyperlipidemia patients showed an increasing trend, and the dynamic balance of cholesterol was disrupted. The present study shows a similar trend of cholesterol changes in hyperlipidemia patients as in other clinical studies. However, relative to healthy controls, only lathosterol (%, increased) and stigmasterol (%, decreased) in the ratio of indicators related to cholesterol metabolism to cholesterol in the hyperlipidemia patients group were statistically different between the two groups (*P* < 0.05). Except for squalene, the concentrations of all indicators were higher in the FHC group than in the hyperlipidemic group (*P* < 0.05). The squalene (%), lathosterol (%), stigmasterol (%), and sitosterol (%) were lower in the FHC group than in the hyperlipidemic group; only desmosterol (%) was higher than the hyperlipidemic group. Although the concentrations of all indicators related to cholesterol metabolism increased in patients with FHC, the proportion of metabolic indicators, except desmosterol, decreased relative to overall cholesterol concentrations. This implies a further disturbance in cholesterol metabolism levels in patients with FHC than in the hyperlipidemic group.

We performed a correlation analysis of lipid-related and cholesterol metabolism indices for all three groups separately. The results showed that the moderate and strong correlations were 32.50% in the healthy group, 31.25% in the hyperlipidemic group, and increased to 76.92% in the FHC group. We suggest that this may be due to the enhanced synergistic effect of lipid factors and cholesterol indicators in patients with FHC. Since FHC belong to an autosomal dominant disorder, abnormal expression of the gene may lead to abnormal metabolism among lipid-related indicators.

Subsequently, we used logistic regression analysis of cholesterol metabolic indicators and baseline data of patients to analyze the etiology of hyperlipidemia disease development. The results showed that glucose, LDL-C, lathosterol, and sitosterol were independent influencing factors for the development of hyperlipidemia, respectively. The logistic regression model had better diagnostic efficacy (AUC = 0.871) and was superior to the diagnostic effectiveness of the four indicators alone. The result is supported by recent study, except for the traditionally available lipid-lowering treatment options, other novel therapies have been shown to favorably impact dense LDL, among them the antidiabetic class of agents [24]. In a word, a more personalized but comprehensive approach is needed instead of a “one-size-fits-all” intervention, different aspects of the diagnosis and therapy of dyslipidemia deserve to be mentioned in the era of contemporary medicine [25]. In the current world, with the COVID-19 pandemic, diabetes, obesity, and cardiovascular disease are related to an increased risk for severe forms of COVID-19 and resulting death, there is a direct effect of COVID-19 on the cardiovascular system and metabolic homeostasis, new markers reflecting the prognosis of cardiovascular disease need to be further explored [26, 27].

## 5. Conclusion

The concentration of cholesterol metabolic markers tended to increase in varying degrees with increasing disease severity; however, the increase in marker concentration did not imply the same trend in their concentration ratio to cholesterol. We suggest that the study of cholesterol synthesis and decomposition markers can serve as a reference index and therapeutic target for related diseases caused by changes in its concentration.

## Figures and Tables

**Table 1:** Comparison of baseline data, cholesterol, blood glucose, blood pressure, and the concentration of the sterols between the three groups.

Variable	Healthy control (*n* = 64)	Hyperlipidemia patients (*n* = 69)	FHC patients (*n* = 25)	*H*/*Z*/*X*^2^	*P*
Age (years)	51 (45, 55)	50.3 ± 5.8	29.5 ± 23	-0.043	0.966
Gender (male/female)	16/48	28/41	13/12	3.6640	0.056
BMI	24.3 (21.7, 27.7)	26.0 ± 3.6	—	-1.837	0.066
HDL-C (mmol/L)	1.42 (1.30, 1.65)	1.31 (1.13, 1.60)	62.37 ± 32.65	44.102	<0.001^∗∗∗^
LDL-C (mmol/L)	3.03 ± 0.53	3.38(3.10, 4.09)	553.70 ± 167.18	54.454	<0.001^∗∗∗^
TG (mmol/L)	1.20 (0.80, 1.40)	2.10 (1.60, 3.50)	108.41 ± 33.86	75.824	<0.001^∗∗∗^
TC (mmol/L)	4.75 ± 0.65	5.35 (4.89, 6.15)	650.86 ± 179.02	57.072	<0.001^∗∗∗^
FPG (mmol/L)	5.00 (4.76, 5.36)	5.00 (4.90, 6.00)	—	-3.487	<0.001^∗∗∗^
SBP (mmHg)	120 (110, 120)	120 (110, 130)	—	-1.766	0.077
DBP (mmHg)	80 (70, 80)	80 (70, 90)	—	-1.258	0.208
Squalene (*μ*mol/L)	2.943 (2.408, 3.698)	3.885 (2.754, 6.180)	5.035 (2.886, 14.184)	12.249	0.002^∗∗^
Squalene/cholesterol (%)	0.797 (0.656, 1.105)	0.887 (0.587, 1.532)	0.434 ± 0.173	39.734	<0.001^∗∗∗^
Desmosterol (*μ*mol/L)	0.618 ± 0.182	0.749 ± 0.214	8.379 ± 3.889	71.156	<0.001^∗∗∗^
Desmosterol/cholesterol (%)	0.161 (0.136, 0.190)	0.169 ± 0.044	0.497 (0.349, 0.796)	52.468	<0.001^∗∗∗^
Lathosterol (*μ*mol/L)	5.362 (4.115, 5.613)	8.325 ± 3.246	14.182 (8.649, 24.603)	48.228	<0.001^∗∗∗^
Lathosterol/cholesterol (%)	1.535 ± 0.584	1.907 ± 0.751	0.989 (0.627, 1.272)	27.472	<0.001^∗∗∗^
Campesterol (*μ*mol/L)	5.296 (3.894, 7.905)	5.242 (3.491, 8.620)	16.150 (9.532, 28.509)	38.999	<0.001^∗∗∗^
Campesterol/cholesterol (%)	1.484 (0.993, 2.339)	1.180 (0.871, 1.927)	1.049 (0.768, 1.625)	6.442	0.040^∗^
Stigmasterol (*μ*mol/L)	1.193 (1.088, 1.344)	1.231 ± 0.278	3.709 (2.469, 5.583)	51.190	<0.001^∗∗∗^
Stigmasterol/cholesterol (%)	0.318 (0.282, 0.387)	0.282 ± 0.070	0.217 (0.165, 0.318)	17.292	<0.001^∗∗∗^
Sitosterol (*μ*mol/L)	7.352 (5.305, 9.383)	8.194 (5.763, 11.956)	19.335 (13.342, 33.245)	42.617	<0.001^∗∗∗^
Sitosterol/cholesterol (%)	2.080 (1.481, 2.505)	1.842 (1.548, 2.449)	1.291 (0.938, 1.741)	17.663	<0.001^∗∗∗^

FHC: familial hypercholesterolemia; HDL-C: high-density lipoprotein cholesterol; LDL-C: low-density lipoprotein cholesterol; TG: total triglyceride; TC: total cholesterol. FPG: fasting plasma glucose; SBG: systolic blood pressure; DBG: diastolic blood pressure. ^∗∗∗^: *P* < 0.001; ^∗∗^: *P* < 0.01; ^∗^: *P* < 0.05.

**Table 2:** The correlation between cholesterol metabolic markers and lipids in health group.

Factor/Cor.	TG	CHO	HDL-C	LDL-C	Squa	Squa (%)	Desm	Desm (%)	Lath	Lath (%)	Camp	Camp (%)	Stig	Stig (%)	Sito	Sitos (%)
TG	1															
CHO	0.034	1														
HDLC	-.322^∗∗^	.307^∗^	1													
LDL-C	0.201	.896^∗∗^	0.068	1												
Squa	0.055	0.150	0.012	0.124	1											
Squa (%)	0.052	0.117	0.010	0.088	.997^∗∗^	1										
Desm	0.216	.438^∗∗^	0.103	.418^∗∗^	-0.216	-0.233	1									
Desm (%)	0.210	-0.132	-0.038	-0.093	-.270^∗^	-.264^∗^	.766^∗∗^	1								
Lath	.531^∗∗^	0.140	-0.177	0.214	-0.014	-0.025	.318^∗^	0.169	1							
Lath (%)	.483^∗∗^	-.283^∗^	-.275^∗^	-0.165	-0.058	-0.054	0.094	.262^∗^	.878^∗∗^	1						
Camp	-.259^∗^	.510^∗∗^	.260^∗^	.416^∗∗^	0.048	0.034	0.147	-0.115	-.250^∗^	-.429^∗∗^	1					
Camp (%)	-.294^∗^	.289^∗^	0.181	0.214	0.028	0.024	0.012	-0.100	-.332^∗∗^	-.400^∗∗^	.950^∗∗^	1				
Stig	-0.023	0.156	-0.036	0.183	0.132	0.116	0.054	-0.030	-0.185	-0.224	0.104	0.071	1			
Stig (%)	-0.030	0.048	-0.079	0.083	0.122	0.111	0.000	-0.001	-0.216	-0.197	0.064	0.063	.991^∗∗^	1		
Sito	-.261^∗^	.437^∗∗^	.273^∗^	.276^∗^	0.093	0.082	-0.018	-0.246	-0.174	-.334^∗∗^	.791^∗∗^	.737^∗∗^	0.074	0.042	1	
Sito (%)	-.301^∗^	0.054	0.141	-0.062	0.059	0.062	-0.231	-0.187	-.292^∗^	-.255^∗^	.643^∗∗^	.715^∗∗^	0.023	0.047	.896^∗∗^	1

%: cholesterol metabolism markers/cholesterol; Squa: squalene (mg/dl); Desm: desmosterol (mg/dl); Lath: lathosterol (mg/dl); Camp: campesterol (mg/dl); Stig: stigmasterol (mg/dl); Sito: sitosterol (mg/dl); Cor: correlation; Sig: significant; TG: triglyceride; CHO: total cholesterol standard values; HDL-C: high-density lipoprotein cholesterol; LDL-C: low-density lipoprotein cholesterol; Glu: glucose. ^∗∗∗:^*P* < 0.001; ^∗∗^: *P* < 0.01; ^∗^: *P* < 0.05.

**Table 3:** The correlation between cholesterol metabolic markers and lipids in hyperlipidemia group.

Factor/Cor.	TG	CHO	HDL	LDL	Squa	Squa (%)	Desm	Desm (%)	Lath	Lath (%)	Camp	Camp (%)	Stig	Stig (%)	Sito	Sitos (%)
Factor/Cor.	1															
TG	.284^∗∗^	1														
CHO	-0.156	.614^∗∗^	1													
HDL	.292^∗∗^	.913^∗∗^	.524^∗∗^	1												
LDL	.417^∗∗^	.189^∗^	-0.001	0.153	1											
Squa	0.068	-.400^∗∗^	-.420^∗∗^	-.407^∗∗^	.608^∗∗^	1										
Squa (%)	.593^∗∗^	.530^∗∗^	.238^∗∗^	.483^∗∗^	.348^∗∗^	-.240^∗∗^	1									
Desm	.544^∗∗^	0.118	0.022	0.113	.286^∗∗^	-0.064	.845^∗∗^	1								
Desm (%)	.633^∗∗^	.261^∗∗^	-0.089	.255^∗∗^	.317^∗∗^	-0.098	.597^∗∗^	.490^∗∗^	1							
Lath	.281^∗∗^	-.351^∗∗^	-.551^∗∗^	-.317^∗∗^	0.036	.404^∗∗^	-0.029	0.108	.532^∗∗^	1						
Lath (%)	0.037	.545^∗∗^	.598^∗∗^	.481^∗∗^	.255^∗∗^	-.280^∗∗^	.357^∗∗^	0.122	0.003	-.587^∗∗^	1					
Camp	-.280^∗∗^	0.117	.338^∗∗^	0.065	0.062	0.130	-.181^∗^	-.275^∗∗^	-.437^∗∗^	-.356^∗∗^	.686^∗∗^	1				
Camp (%)	.227^∗∗^	.404^∗∗^	.384^∗∗^	.349^∗∗^	.198^∗^	-.268^∗∗^	.462^∗∗^	.347^∗∗^	.176^∗^	-.387^∗∗^	.515^∗∗^	0.117	1			
Stig	-.166^∗^	-.371^∗∗^	-0.112	-.398^∗∗^	-0.137	.284^∗∗^	-.307^∗∗^	-0.098	-.332^∗∗^	0.045	-0.134	.225^∗∗^	.373^∗∗^	1		
Stig (%)	0.116	.600^∗∗^	.593^∗∗^	.503^∗∗^	.246^∗∗^	-.304^∗∗^	.369^∗∗^	0.096	0.101	-.513^∗∗^	.891^∗∗^	.524^∗∗^	.576^∗∗^	-0.107	1	
Sito	-.318^∗∗^	0.003	.214^∗∗^	-0.083	-0.033	.221^∗∗^	-.326^∗∗^	-.388^∗∗^	-.461^∗∗^	-.199^∗^	.421^∗∗^	.809^∗∗^	0.079	.380^∗∗^	.505^∗∗^	1

^∗∗∗^: *P* < 0.001; ^∗∗^: *P* < 0.01; ^∗^: *P* < 0.05.

**Table 4:** The correlation between cholesterol metabolic markers and lipids in the familial hypercholesteremia group.

Factor/Cor.	TG	CHO	HDLC	LDL-C	Squa	Squa (%)	Desm	Desm (%)	Lath	Lath (%)	Camp	Camp (%)	Stig	Stig (%)	Sito	Sito (%)
TG	1															
CHO	0.193	1														
HDLC	-0.177	0.141	1													
LDL-C	0.172	.982^∗∗^	0.012	1												
Squa	0.452	0.207	-0.241	0.283	1											
Squa (%)	0.292	-0.450	-0.261	-0.378	.601^∗∗^	1										
Desm	0.171	0.203	0.038	0.212	.466^∗^	0.054	1									
Desm (%)	-0.019	-0.466	0.238	-.533^∗^	-.565^∗∗^	-0.124	0.034	1								
Lath	0.262	0.068	0.013	0.086	.512^∗∗^	0.395	.611^∗∗^	-0.144	1							
Lath (%)	-0.030	-0.330	-0.032	-0.321	-0.295	0.185	-0.013	.555^∗∗^	.415^∗^	1						
Camp	0.324	-0.140	0.008	-0.114	.469^∗^	.449^∗^	0.070	-0.114	-0.084	-.400^∗^	1					
Camp (%)	0.078	-0.423	0.045	-0.438	-0.146	0.266	-0.233	0.316	-0.282	-0.138	.730^∗∗^	1				
Stig	0.386	-0.141	-0.137	-0.125	0.368	0.396	-0.038	-0.022	-0.213	-.452^∗^	.909^∗∗^	.795^∗∗^	1			
Stig (%)	0.087	-0.380	0.028	-0.399	-0.197	0.221	-0.271	0.378	-0.336	-0.132	.675^∗∗^	.986^∗∗^	.790^∗∗^	1		
Sito	0.207	-0.308	-0.029	-0.300	0.136	0.371	-0.116	0.125	-0.211	-0.277	.895^∗∗^	.950^∗∗^	.924^∗∗^	.922^∗∗^	1	
Sito (%)	0.071	-0.409	0.025	-0.423	-0.163	0.250	-0.235	0.321	-0.281	-0.132	.708^∗∗^	.998^∗∗^	.788^∗∗^	.989^∗∗^	.944^∗∗^	1

^∗∗∗^: *P* < 0.001; ^∗∗^: *P* < 0.01; ^∗^: *P* < 0.05.

**Table 5:** Application of logistic regression analysis in the diagnosis of hyperlipidemia.

Detection factor	*B*	*P*	Exp (*B*)	95% CI of EXP (*B*)	
				LL	UL
Gender (1)	-0.698	0.200	0.497	0.171	1.446
Age (years)	0.025	0.492	1.025	0.955	1.100
BMI (kg/m^2^)	-0.070	0.312	0.932	0.814	1.068
Glucose (mmol/L)	0.794	0.037^∗^	2.213	1.049	4.669
HDL-C (mmol/L)	-0.640	0.155	0.527	0.218	1.273
LDL-C (mmol/L)	1.448	0.002^∗∗^	4.255	1.676	10.802
Squalene (*μ*mol/L)	-0.153	0.616	0.859	0.473	1.558
Desmosterol (*μ*mol/L)	-21.273	0.579	<0.001	<0.001	2.593^∗^10^24^
Lathosterol (*μ*mol/L)	11.362	0.001^∗∗^	8.599^∗^10^5^	116.183	6.364^∗^10^8^
Campesterol (*μ*mol/L)	-4.205	0.238	0.015	<0.001	16.203
Stigmasterol (*μ*mol/L)	-1.481	0.828	0.227	<0.001	1.404^∗^10^6^
Sitosterol (*μ*mol/L l)	7.821	0.021^∗^	2492.153	3.216	1.931^∗^10^6^
Constant	-10.921	0.001	<0.001		

**Table 6:** The result of the ROC curve.

Factor	AUC	SE	95% CI	*z*	*P*	Youden index	Associated criterion	Sensitivity (%)	Specificity (%)
Glucose (mmol/L)	0.711	0.044	0.626-0.787	4.793	<0.001	0.318	>3.81	33.33	98.44
LDL-C (mmol/L)	0.675	0.047	0.589-0.754	3.752	<0.001	0.323	>5.15	66.67	65.62
Lathosterol (*μ*mol/L)	0.753	0.042	0.670-0.824	6.004	<0.001	0.444	>0.26	64.71	79.69
Sitosterol (*μ*mol/L)	0.587	0.050	0.498-0.672	1.753	0.080	0.216	>0.46	29.41	92.19
Model	0.871	0.030	0.801-0.923	12.459	<0.001	0.601	>0.433	85.07	75.00

AUC: area under curve; SE: standard error.

## Data Availability

All data associated with this study are present in the paper, and all figures and tables have associated raw data. Any materials that can be shared will be released via a material transfer agreement. Written informed consent was obtained from each patient. At the same time, it was supported by the Ethics Committee of Anzhen Hospital.

## References

[1] Wang S., Xu L., Jonas J. B., You Q. S., Wang Y. X., Yang H. (2011). Prevalence and associated factors of dyslipidemia in the adult Chinese population. *PLoS One*.

[2] Blom W. A., Koppenol W. P., Hiemstra H., Stojakovic T., Scharnagl H., Trautwein E. A. (2019). A low-fat spread with added plant sterols and fish omega-3 fatty acids lowers serum triglyceride and LDL-cholesterol concentrations in individuals with modest hypercholesterolaemia and hypertriglyceridaemia. *European Journal of Nutrition*.

[3] Moore K. J., Sheedy F. J., Fisher E. A. (2013). Macrophages in atherosclerosis: a dynamic balance. *Nature Reviews Immunology*.

[4] Le Roy T., Lécuyer E., Chassaing B. (2019). The intestinal microbiota regulates host cholesterol homeostasis. *BMC Biology*.

[5] Vekic J., Zeljkovic A., Cicero A. F. G. (2022). Atherosclerosis development and progression: the role of atherogenic small, dense LDL. *Medicina*.

[6] Gojkovic T., Vladimirov S., Spasojevic-Kalimanovska V. (2017). Can non-cholesterol sterols and lipoprotein subclasses distribution predict different patterns of cholesterol metabolism and statin therapy response?. *Clinical Chemistry and Laboratory Medicine*.

[7] Wu A. H. (2014). Biomarkers for cholesterol absorption and synthesis in hyperlipidemic patients: role for therapeutic selection. *Clinics in Laboratory Medicine*.

[8] Baila-Rueda L., Cenarro A., Lamiquiz-Moneo I. (2018). Cholesterol oversynthesis markers define familial combined hyperlipidemia _versus_ other genetic hypercholesterolemias independently of body weight. *The Journal of Nutritional Biochemistry*.

[9] Tromp T., Hartgers M., Hovingh G., Blom D., Cuchel M., Raal F. (2021). Worldwide perspective on homozygous familial hypercholesterolemia diagnosis, treatment and outcome - results from the HICC registry. *Atherosclerosis*.

[10] Garcia-Otin A., Cofán M., Junyent M. (2007). Increased intestinal cholesterol absorption in autosomal dominant hypercholesterolemia and no mutations in the low-density lipoprotein receptor or apolipoprotein B genes. *The Journal of Clinical Endocrinology & Metabolism*.

[11] Di Ciaula A., Wang D. Q.-H., Portincasa P. (2018). An update on the pathogenesis of cholesterol gallstone disease. *Current Opinion in Gastroenterology*.

[12] Alphonse P. A., Jones P. J. (2016). Revisiting human cholesterol synthesis and absorption: the reciprocity paradigm and its key regulators. *Lipids*.

[13] Santosa S., Varady K. A., Abu Mweis S., Jones P. J. (2007). Physiological and therapeutic factors affecting cholesterol metabolism: does a reciprocal relationship between cholesterol absorption and synthesis really exist?. *Life Sciences*.

[14] Caponio G. R., Wang D. Q.-H., Di Ciaula A., De Angelis M., Portincasa P. (2020). Regulation of cholesterol metabolism by bioactive components of soy proteins: novel translational evidence. *International Journal of Molecular Sciences*.

[15] Mori K., Ishida T., Tsuda S. (2017). Enhanced impact of cholesterol absorption marker on new atherosclerotic lesion progression after coronary intervention during statin therapy. *Journal of Atherosclerosis and Thrombosis*.

[16] Miettinen T., Gylling H., Nissinen M. (2011). The role of serum non-cholesterol sterols as surrogate markers of absolute cholesterol synthesis and absorption. *Nutrition, Metabolism, and Cardiovascular Diseases*.

[17] Schaefer L. E., Nechemias C. (1965). Endogenous hormones, lipid metabolism, and coronary artery disease. *Progress in Cardiovascular Diseases*.

[18] Paramsothy P., Knopp R. H., Kahn S. E. (2011). Plasma sterol evidence for decreased absorption and increased synthesis of cholesterol in insulin resistance and obesity. *The American Journal of Clinical Nutrition*.

[19] Silbernagel G., Chapman M. J., Genser B. (2013). High intestinal cholesterol absorption is associated with cardiovascular disease and risk alleles in _ABCG8_ and _ABO_ : evidence from the LURIC and YFS cohorts and from a meta-analysis. *Journal of the American College of Cardiology*.

[20] Group SSSS (1994). Randomised trial of cholesterol lowering in 4444 patients with coronary heart disease: the Scandinavian Simvastatin Survival Study (4S). *The Lancet*.

[21] Vekic J., Zeljkovic A., Al Rasadi K. (2022). A new look at novel cardiovascular risk biomarkers: the role of atherogenic lipoproteins and innovative antidiabetic therapies. *Metabolites*.

[22] Rogacev K. S., Pinsdorf T., Weingärtner O. (2012). Cholesterol synthesis, cholesterol absorption, and mortality in hemodialysis patients. *Clinical Journal of the American Society of Nephrology*.

[23] Hoenig M. R., Sellke F. W. (2010). Insulin resistance is associated with increased cholesterol synthesis, decreased cholesterol absorption and enhanced lipid response to statin therapy. *Atherosclerosis*.

[24] Ali A. (2021). Lipoproteins and cardiovascular disease: an update on the clinical significance of atherogenic small, dense LDL and new therapeutical options. *Biomedicine*.

[25] Fras Z., Jug B., Penson P. E., Rizzo M. (2021). Challenges and opportunities on lipid metabolism disorders diagnosis and therapy: novel insights and future perspective. *Metabolites*.

[26] Mahmeed W. A., Al-Rasadi K., Banerjee Y. (2021). Promoting a Syndemic approach for Cardiometabolic disease management during COVID-19: the CAPISCO international expert panel. *Frontiers in Cardiovascular Medicine*.

[27] Rizvi A. A., Janez A., Rizzo M. (2021). Cardiometabolic alterations in the interplay of COVID-19 and diabetes: current knowledge and future avenues. *International Journal of Molecular Sciences*.

